# Books and Authors

**Published:** 1911-01

**Authors:** 


### BOOKS AND AUTHORS

**The Practical Medicine Series.** Ten volumes. Under the general editorial charge of **Gustavus P. Head,** M.D., Professor of Laryngology and Rhinology in the Chicago Post-Graduate Medical School. Vol. I, General Medicine. Vol. II, General Surgery. Vol. III, Eye, Ear, Nose and Throat. Vol. IV, Gynecology. Vol. V, Obstetrics. Vol. VI, General Medicine. Series 1910. Chicago: The Year Book Publishers. (Entire series, $10.00. Single copies sold separately at $1.25 to $2.00 each.)

I.—*General Medicine.*—Billings and Salisbury have gleaned from the year's literature many valuable hints concerning internal medicine, relating in particular to diseases of the respiratory organs and of the circulatory organs, these two sub-topics occupying the greater portion of the book. One of the important sections is Forchheimer's treatment of chronic bronchitis, especially important being what he says relating to "catching cold." Arteriosclerosis, one of the most absorbing of the bloodvessel diseases, is dealt with by various authors whose views have been abstracted, and which should be read by every physician who practises internal medicine. Other topics of importance, including infectious diseases, metabolic diseases, diseases of the ductless glands, and diseases of the kidneys are dealt with in this number.

II.—*General Surgery.*—Murphy continues to report the literature of the year for this volume. In his introduction he makes several interesting statements, one of which is that "fulminating," "explosive," and "initially fatal" cases of peritonitis are gone, so far as the informed physician is concerned. And he might have added, while keeping strictly within the bounds of truth, that their banishment is the result, very largely, at least, of his own teaching. That there is a renaissance of the surgery of the bones and joints is another fact for which Murphy is largely responsible, having contributed to it by insisting through clinics and society addresses upon better methods of diagnosis and treatment. We are pleased to read a little further on that the number of "exploratory" incisions is lessening, due, of course, to better diagnostic acumen on the part of operators. A few surgeons have long understood this fact, but it has not been generally admitted until lately. Murphy's introduction is full of valuable thought and might be read with profit by every man who even makes a practice of doing operations of any kind.

Surgery of the bloodvessels is attracting a good deal of attention nowadays and some abstracts, well illustrated, are published. Goiter is dealt with in several pages and some good illustrations. Gastrointestinal surgery, the appendix, and hernia receive large space and copious illustration, as might be expected; while the kidneys and bladder are well handled. The entire volume is full of interest and is one of the better indexes of progress in surgery for the year 1909.

III.—*Eye, Ear, Nose, and Throat.*—Progress pertaining to the eye, compiled by Casey A. Wood, occupies about one-half the pages of this book, owing to the great wealth of the year 1909 in ophthalmic literature. From it all, however, Wood has made a judicious selection, presenting in condensed fashion that which will prove of most value to everyday practitioners. The material relating to the ear is set forth by Albert H. Andrews, who brings out the essentials of the year in readable form. One important feature, never to be forgotten by the family doctor, is the liability of aural complications to follow in the trail of the exanthemata,—well touched upon here.

The nose and throat are presented by the editor, Dr. Head. Sluder's operation for opening the maxillary antrum is described and Darling's cytologic examinations of the discharge in eighty cases of suppuration of the maxillary sinus are stated in the form of conclusions. Hay fever is presented interestingly, while the whole topic embraced in this abstract relating to nose and throat has been made with exceeding care as well as with good judgment.

IV.—*Gynecology.*—Dudley continues as reporter for this volume, and the material he presents is excellent. Bachelle is associated with him in its preparation. This is a splendid review of the progress in gynecology and will prove helpful to everybody who reads it. We note that Hayd (Buffalo) is quoted in regard to prolapse of the uterus (p. 179) as preferring to remove the uterus rather than to fix it to the abdominal wall, stating reasons therefor. Hayd's operation for rectocele (BUFFALO MEDICAL JOURNAL, January, 1910) and ruptured perineum (p. 187) is also described in detail and the illustrations are reproduced. Reed (Cincinnati) has originated a method of dealing with the painful uterus which here (p. 217) is described. This is employed where there is no organic disease but merely a neurosis. An analgesic, according to Reed's formula, is employed.

V.—*Obstetrics.*—De Lee and Stowe are to be credited very justly with an admirable resume of new observations gleaned from the literature of 1909 pertaining to pregnancy, labor, the puerperium, and the newborn. Eclampsia claims considerable attention, though nothing new of real value has been evolved. In competent hands the compiler believes that immediate delivery is to be adhered to as offering the best chance to the woman. Obstetricians of the greater experience will agree with him in general. Obstetric anesthesia and obstetric operations, especially Cesarean section, and the obstetric hemorrhages receive marked attention. The puerperium and puerperal sepsis, the latter in particular, are presented in detail, while the newborn is considered with some care. We have been much interested in De Lee's comments, inclosed in brackets, scattered throughout the book, accentuating this point and that either in approval or disapproval, which increase the value of many of the selections.

VI.—*General Medicine.*—This appears to be the second volume by Billings and Salisbury published this year. We have noticed the first and now take up the second. Infectious diseases occupy nearly one hundred pages, typhoid fever taking up almost half the space. All the known methods of diagnosis are considered; treatment, too, is dealt with, but when all is said and done very little use of drugs should be made in typhoid, diet being the main reliance, through its proper regulation. The history of diet, though, shows a steady tendency toward increase in quantity to meet the waste of the disease. The absurd prejudice against milk in typhoid is confuted by Coleman in this abstract. If in some cases milk curds appear in the stools the milk should be peptonised or diminished in quantity. Diseases of the stomach occupy nearly ninety pages, in the course of which Dr. Charles G. Stockton's article on gastric ulcer is abstracted, giving his views as to the etiology of this lesion. Diseases of the intestines form a considerable portion of the book, also about ninety pages, in which many questions relating to diagnosis and treatment are discussed.

The six books we here have noticed form a splendid compend of the literature of medicine for a year (1909) and afford an economic way for the busy doctor to keep himself well informed as to the progress in medicine during that period.

**Treatise on Diseases of the Skin. By Henry W. Stelwagon,** M.D., Ph.D., Professor of Dermatology in Jefferson Medical College, Philadelphia. Sixth edition. Octavo, pp. 1195. Illustrated. Philadelphia and London: W. B. Saunders Co. 1910. Cloth. $6.00, net).

Stelwagon has stood in the forefront of dermatological teachers and authors for many years. His treatise, now in its sixth edition, appeared first in 1902, and has been reviewed in these columns from time to time since the beginning. The book is presented in this edition with an elimination of obsolete and unnecessary material, thus permitting an addition of much new and more important literature. Hence, in addition to tropical skin affections, which appeared in the last previous edition, we find here granuloma annulare and lichen nitidus. Sporotrichosis, a disease growing in importance, is presented for the first time in this issue. Grainmite dermatitis, or grain itch, as popularly known, is given detailed description and is well illustrated. The brown-tail moth dermatitis also receives due attention. Pellagra now arresting the attention of observers all over the country, is given considerable space and the experience of many American dermatologists is recorded. The illustrations of pellagra are graphic. Foot notes, references and excellent illustrations, twenty-five of which are new, added to the splendid text, go to make up one of the better of the many excellent dermatological textbooks.

**The Principles of Pathology.** Volume I, **General Pathology.** By **J. George Adami,** M.A., M.D., LL.D., F.R.S., Professor of Pathology in McGill University, Montreal. Second edition, thoroughly revised. Octavo, 1027 pages, with 329 engravings and 18 plates. Lea & Febiger, Publishers: Philadelphia and New York. 1910. (Cloth, $6.00 net.)

It is scarce two years since we wrote concerning the first edition of this great work. The second edition, is in demand within an unusually brief period of time for such an elaborate work on this topic. This bespeaks the fact that pathology is beginning to take hold of student and practitioner; in other words, that teachers are beginning to instruct in this subject in a manner that impresses its importance, that fixes it definitely in the mind of the learner as lying at the base of the knowledge of disease. Another point brought out is, that pathology cannot be learned as it should be from elementary books, but only from a treatise that practically exhausts our present knowledge of the subject. Such a treatise is offered by Professor Adami in this work. He presents the subject in a comprehensive form, but so attractively that every one may learn even though he runs.

It is obviously out of place to undertake an analytical review of such a treatise at this time, it being extremely doubtful if such a discourse would command attention; but we may say a few words about the book and its author in the expectation that some good may come of it. Adami is generally recognised as one of the foremost pathologists of the day, one, too, who, while mastering his subject, is able to impart his knowledge to others through a literary style that is attractive and a method that not only fixes the attention but fastens its essentials upon the memory.

In this second edition Professor Adami has made many improvements, though the general appearance and size of the first volume remains as before. Some rearrangement is manifest, some of the sections have been rewritten, and some of the later developments have been incorporated; for example, the ultramicroscopic causes of disease appears as new material. It may be stated as a general fact that almost every topic has been revised, bringing the whole subject of general pathology forward to the very date the book was issued. We have said already that we did not propose in this notice to enter into an extended review, but we think the chapters on inheritance should be read by every physician who cares to be well informed. They help to clear up many doubtful points relating to this complex and even, in some respects, moot problem. Adami deals with the subject in a most attractive manner, and what he says will repay well the time spent in reading these chapters. The next volume will present systemic pathology for consideration.

---

**The Physician's Visiting List** (Lindsay and Blakiston's) **for 1911.** Philadelphia: P. Blakiston's Son & Co., 1012 Walnut Street.

The proud heritage of sixty years' service belongs to this excellent visiting list and diary for physicians. It has stood the test of time and presents itself for the year 1911, in splendid form to make the campaign for the first year of the second decade of the twentieth century. It contains all the clinical memoranda needed at the bedside, such as calendars for 1911 and 1912; obstetric table; incompatibilities, poisoning antidotes and methods of treatment; metric system and table for converting apothecary's weights and measures; dose table; asphyxia and apnea hints; thermometric comparisons; together with blank leaves for visiting list, addresses, accounts, engagements, records, and many other needs of the busy doctor. It is well to remember that only such books of value outlive the early years of their birth, and this is the only one of its kind that now launches forth upon its sixtieth year, and that in the full vigor of a young manhood. The publishers may justly take great pride in this sturdy, useful, and beautiful book.

---

**An Anatomical and Surgical Study of Fractures of the Lower end of the Humerus.** By **Astley Paston Cooper Ashurst,** A.B., M.D., Prosector of Applied Anatomy in the University of Pennsylvania. The Samuel D. Gross Prize Essay of the Philadelphia Academy of Surgery, 1910. Octavo, pp. 163. Illustrated. Philadelphia and New York: Lea & Febiger. 1910. (Price, $2.75.)

This study relating to fractures of the elbow joint, depicting the anatomy and surgery of the structures involved, received the Samuel D. Gross Prize of the Philadelphia Academy of Surgery for 1910, amounting to fifteen hundred dollars. It is illustrated by 150 skiagraphs, half-tones, and drawings made from living patients and dead subjects, the whole forming the most complete exposition of bone injury of the elbow yet put forth for the inspection and benefit of surgeons. The book is imperial octavo in size and is printed on heavy calendered book paper, thus enabling the pictures to be brought out to the best advantage. In establishing the fund from which this prize has been awarded Professor Gross provided that it should be allotted every five years to the writer of the best original essay illustrative of some subject in surgical pathology or practical surgery. The committee of award in this instance was made up of the trustees of the fund, Drs. William J. Taylor, Richard H. Harte, and De Forrest Willard. Every one who reads the book will agree that the award is bestowed with propriety. Every surgeon who deals with surgical injuries needs the monograph.

**Medical Chaos and Crime.** By **Norman Barnesby,** M.D. Octavo, pp. 384. London and New York: Mitchell Kennerly. 1910.

On the front cover it is stated that this book is, "An inquiry into the widespread demoralisation of the medical profession, a warning to the victimised public, and an earnest plea for immediate and drastic reform." The author says he offers testimony that will quickly convince the openminded reader, whether layman or physician, that a terrible problem confronts this nation, the neglect of which means such a wanton destruction of health and happiness and such an appalling loss of human life that one recoils from the spectacle. It is a book that will serve no useful purpose; it does not even state conditions fairly. Aside from a ma s of garbled reports and half sentences quoted, the writer offers no testimony that warrants any such sweeping denunciation of the profession of which he claims to be a member. In view of the

**Transactions of the Seventh Annual Conference of State and Territorial Health Officers** with the United States Public Health and Marine Hospital Service, at Washington, D. C., June 2, 1910.

These annual conferences of the state and territorial health officers held under the auspices of the United States Public Health and Marine Hospital Services are fraught with much good. It is important to secure uniformity of administration as far as possible all over the country in relation to matters that concern the public health. This book records the proceedings of the seventh conference held June 2 and 3, 1909, at the bureau of Public Health and Marine Hospital Service at Washington, presided over by Surgeon General Wyman. Health officers from twenty-seven states and territories were present. Among the subjects discussed were rabies, leprosy, typhoid fever, hookworm disease, and pellagra. The transactions are full of interest, the debates being instructive and well conducted.

**The Physicians' Pocket Account Book,** by **J. J. Taylor,** M.D., 212 pages. Leather. Price, $1.00 postpaid. J. J. Taylor, Publisher, 4105 Walnut Street, Philadelphia, Pa.

A compact account, like this one, is useful for the desk or pocket. It is, moreover, a time-saving device, and will prove an economic investment. Having it at hand entries will be made which, otherwise, might be overlooked. Practical directions and hints are given in the book that also will prove of value in many instances. It is one of the better books of its kind.

